# Evaluation of a cluster-randomized controlled trial: Communities for Healthy Living, family-centered obesity prevention program for Head Start parents and children

**DOI:** 10.1186/s12966-022-01400-2

**Published:** 2023-01-11

**Authors:** Cristina Gago, Alyssa Aftosmes-Tobio, Jacob P. Beckerman-Hsu, Carly Oddleifson, Evelin A. Garcia, Kindra Lansburg, Roger Figueroa, Xinting Yu, Nicole Kitos, Merieka Torrico, Jessie Leonard, Janine K. Jurkowski, Josiemer Mattei, Erica L. Kenney, Sebastien Haneuse, Kirsten K. Davison

**Affiliations:** 1 Department of Nutrition, Harvard T.H. Chan School of Public Health, Boston, MA 02115 USA; 2 School of Social Work, Boston College, 140 Commonwealth Ave, 115 McGuinn Hall, Chestnut Hill, MA 02467 USA; 3 Department of Global Health & Population, Harvard T.H. Chan School of Public Health, Boston, MA 02115 USA; 4 Action for Boston Community Development (ABCD), Boston, MA 02111 USA; 5 Division of Nutritional Sciences, College of Human Ecology, Cornell University, Ithaca, NY 14853 USA; 6 Community Action Agency of Somerville (CAAS), Somerville, MA 02143 USA; 7 Department of Health Policy, Management, and Behavior, State University of New York, Albany, NY 12222 USA; 8 Department of Biostatistics, Harvard T.H. Chan School of Public Health, Boston, MA 02115 USA

**Keywords:** Empowerment, Early childhood, Childhood obesity, Obesity prevention trial, Community-based participatory research, Family-centered intervention, Child weight-related behaviors, Parenting practices

## Abstract

**Background:**

This study reports the outcomes of Communities for Healthy Living (CHL), a cluster randomized obesity prevention trial implemented in partnership with Head Start, a federally-funded preschool program for low-income families.

**Methods:**

Using a stepped wedge design, Head Start programs (*n* = 16; Boston, MA, USA) were randomly assigned to one of three intervention start times. CHL involved a media campaign and enhanced nutrition support. Parents were invited to join Parents Connect for Healthy Living (PConnect), a 10-week wellness program. At the beginning and end of each school year (2017-2019), data were collected on the primary outcome of child Body Mass Index z-score (BMIz) and modified BMIz, and secondary outcomes of child weight-related behaviors (diet, physical activity, sleep, media use) and parents’ weight-related parenting practices and empowerment. Data from 2 years, rather than three, were utilized to evaluate CHL due to the COVID-19 pandemic. We used mixed effects linear regression to compare relative differences during intervention vs. control periods (*n* = 1274 vs. 2476 children) in (1) mean change in child BMIz and modified BMIz, (2) the odds of meeting child health behavior recommendations, (3) mean change in parenting practices, and (4) mean change in parent empowerment. We also compared outcomes among parents who chose post-randomization to participate in PConnect vs. not (*n* = 55 vs. 443).

**Results:**

During intervention periods (vs. control), children experienced greater increases in BMIz and modified BMIz (*b* = 0.06, 95% CI = 0.02,0.10; *b* = 0.07, 95% CI = 0.03, 0.12), yet were more likely to meet recommendations related to three of eight measured behaviors: sugar-sweetened beverage consumption (i.e., rarely consume; Odds Ratio (OR) = 1.5, 95% CI = 1.2,2.3), water consumption (i.e., multiple times per day; OR = 1.6, 95% CI = 1.2,2.3), and screen time (i.e., ≤1 hour/day; OR = 1.4, 95% CI = 1.0,1.8). No statistically significant differences for intervention (vs. control) periods were observed in parent empowerment or parenting practices. However, parents who enrolled in PConnect (vs. not) demonstrated greater increases in empowerment (*b* = 0.17, 95% CI = 0.04,0.31).

**Conclusions:**

Interventions that emphasize parent engagement may increase parental empowerment. Intervention exposure was associated with statistically, but not clinically, significant increases in BMIz and increased odds of meeting recommendations for three child behaviors; premature trial suspension may explain mixed results.

**Trial registration:**

ClinicalTrials.gov, NCT03334669, Registered October 2017.

**Supplementary Information:**

The online version contains supplementary material available at 10.1186/s12966-022-01400-2.

## Introduction

The rising prevalence of childhood obesity is well-documented [1, 2], as are the striking disparities that exist in weight status as early as kindergarten, by race, ethnicity, and income [1, 3–5]. Children who develop obesity in childhood carry increased risk of early morbidity and mortality across the lifespan [6], underscoring the need for effective and early prevention efforts [7]. Early intervention is necessary [8], as are programs which effectively engage parents as agents of change in child health promotion [9–11].

Consequently, programs for younger children (i.e., those under five) are gaining momentum in the early care and education (ECE) context [12–14]. With over 80% of 3-5-year-olds in the U.S. attending center-based care [15], ECE serves as a natural space for interventions to reach families with young children [16]. Common intervention strategies often include, for example, the adaptation of ECE curriculum, activities, modelling, and environment [17]. Though ECE-based interventions continue to grow in number and demonstrate promise [16], few directly involve parents as intervention recipients [18–22], beyond children and ECE staff, as there are notable challenges to doing so [22, 23]. This, however, contrasts an abundance of literature evidencing the importance of family contexts and parenting behaviors in obesity prevention [24–26].

In response, our research team partnered with parents and staff of Head Start, a federally-funded school readiness program for young children of low-income U.S. households [27–29], to design and implement Communities for Healthy Living (CHL): an innovative childhood obesity prevention program. Informed by empowerment theories [30–32] and the Family Ecological Model [33], CHL was designed to encourage healthy weight parenting practices [5, 8, 34–36], as a means of promoting healthy child growth and development [37], while recognizing the broader impact of contextual factors, including the social determinants of health, on parenting. Accordingly, we hypothesized that changes in parents’ reported empowerment to shape their family’s social environment and parents’ health-related parenting practices would result in positive health behavior changes among children, which subsequently manifest in the maintenance of healthy weight status.

Aligned with CHL’s theory of change, in this study we evaluate whether children in the intervention vs. the control experienced greater improvements in Body Mass Index z-score (BMIz) and weight-related behaviors (i.e., diet, physical activity, sleep, media use). We also examine whether parents experienced greater improvements in healthy weight parenting practices and empowerment for child health promotion during the intervention period relative to the control.

## Methods

### Intervention

CHL was a cluster randomized family-centered obesity prevention trial implemented in partnership with Head Start in Boston, MA, USA (2016-2019). A detailed description of CHL’s study protocol, including its theoretical background, participatory methods, intervention components and evaluation protocol, is presented elsewhere [37]. The Ethics Review Boards at Harvard University and Boston College approved this study; the trial was registered at ClinicalTrials.gov (NCT03334669; First submitted October 10, 2017 and first posted November 7, 2017). Intervention reporting in this study aligns with the template for intervention description and replication (TIDieR) guide [38]; a populated checklist is included as a supplement (Additional file 3).

Grounded in a Community-Based Participatory Research (CBPR) [39–41] approach [37, 42], CHL content development, implementation, and dissemination were driven by a community-researcher partnership [43]. Briefly, at the start of the trial, a Community Advisory Board (CAB) of Head Start parents and staff was convened to adapt study materials from the 2009 pilot study conducted in upstate New York [44] to ensure a cultural match with the needs of Head Start families in Greater Boston [37]. A full school year (2016-2017) was dedicated to this thorough adaptation process and to pilot testing the measures prior to intervention implementation. Throughout implementation, financial resources were shared by research and Head Start partners via subcontracts. CHL coordinators were hired, using subcontract funds, to work within Head Start agencies to support intervention fidelity, participant recruitment and organizational capacity for data collection and compilation.

The CHL intervention included enhanced nutrition support, a media campaign and an empowerment-focused parenting program. All activities focused on four health-related behaviors in children including diet, physical activity, sleep and media use along with a healthy body weight. While nutrition support services, the provision of health promotion information and programming for parents are part of standard practice at Head Start, CHL enhanced each of these activities. For each intervention component, relevant existing Head Start infrastructure and practices, novel CHL intervention innovations introduced, and the theoretical basis for component development are described in Table 1. The hypothesized pathway by which these intervention components map onto child and parent health, behavior, and empowerment outcomes has been published previously [37].

**Table 1:** Overview of existing Head Start infrastructure, novel CHL intervention innovations introduced, and the theoretical basis for CHL intervention component development

Existing Head Start Infrastructure	Novel CHL Intervention Innovations Introduced	Theoretical Basis for CHL Intervention Component Development
**Intervention Component 1: Enhanced Nutrition Suppor**t		
• Mandated biannual measurement of child height and weight by Head Start staff	• Standardized procedures for height and weight measurements and nutritional counseling ° Developed a technical manual for anthropometric measurement and questionnaire administration ° Created visual aids to facilitate questionnaire administration. ° Delivered ongoing staff training.	• Empowerment Theory [30–32]: Offer staff opportunities for growth and improve Head Start’s capacity for communication
• Child health screening reports sent home biannually	• Revised child health screening reports sent home to more clearly communicate the meaning of BMI, highlight potential next steps for parents, and identify Head Start staff to contact with questions	• Family Ecological Model [45, 46]: Increase parent knowledge regarding child health and parent readiness in identifying relevant support networks
**Intervention Component 2: Media Campaign**		
• Healthy habits brochures and flyers created within programs by Head Start nutritionists; inconsistently delivered and non-standardized	• Redesigned and distributed brochures and flyers for all participating Head Start programs based on themes identified as important by the Community Advisory Board. • Shared community-specific information and resources through online platforms • Created a novel online neighborhood resource map, made publicly available and free to use	• Empowerment Theory [30–32]: Provide parents with the knowledge and skills necessary to identify and utilize local resources • Family Ecological Model [45, 46]: Enhance existing communication strategies and collaboratively develop new educational materials for distribution
**Intervention Component 3: Opt-In Parenting Program (PConnect)**		
• Preexisting programs for parent to engage, learn, and self-reflect (e.g., “Parenting Journey”)	• Developed and implemented a new health- and empowerment-focused program, as a complement to preexisting Head Start parenting programs • 10 two-hour sessions co-led by a Head Start staff member and parent • Online supplements distributed via Facebook	• Empowerment Theory [30–32]: Enhance parent knowledge, self-efficacy, and skills • Family Ecological Model [45, 46]: Address multi-level determinants of child and parent health outcomes

Briefly, CHL enhanced nutrition support services were offered at Head Start to improve staff communication skills, increase parent knowledge of healthy living and link parents with resources valuable for child health promotion. Such enhancements included revised biannual health letters (reviewed and approved by a CAB in the study development stages) which communicated the results of child health screenings, continued training for Head Start nutritionists and standardized protocols and nutrition counseling resources [37]. The media campaign included online and print resources focused on the targeted child health behaviors including brochures and flyers. Tailored to Head Start families, these resources were designed to promote parent knowledge of healthy diet, physical activity, sleep, and media behaviors in children, and to outline policies and practices utilized by Head Start to support child health behaviors.

To promote health-related parental empowerment [30, 32, 47–51], CHL invited parents each spring (January - April) to enroll in an intensive 10 session parenting program (Parents Connect for Healthy Living, or PConnect). Program sessions were co-led by a Head Start parent and staff member. Grounded in empowerment theories [30, 32, 48–51], PConnect was designed to promote the skills parents needed to identify social determinants of child health and access both the relationships and resources necessary for child health promotion [42, 47]. The sessions addressed topics that were important to families, as highlighted by the CAB, and included, for example, healthy family relationships, child personality, neighborhoods and social networks, and parental advocacy [37].

### Trial design

We evaluated the effectiveness of CHL using a pragmatic cluster-randomized controlled trial design. Our reporting of the trial aligns with the Consolidated Standards of Reporting Trials (CONSORT) 2010 recommendations [52]; a populated checklist with the extension for cluster randomized design is included as a supplement (Additional file 2). In 2015, 16 Head Start programs in Greater Boston, serving over 1650 preschool-aged children and their families a year, were recruited to participate [37]. We conducted a statistical power analysis a priori using the Hussey and Hughes approach for mixed effects models within cluster-randomized trials [53].

Using a stepped wedge design [53, 54] the Head Start programs were randomly assigned by the study’s data manager, and with oversight from the study statistician, to one of three intervention start times; this ensured that each program/cluster received the intervention at some point, a more equitable approach (that a waitlist control) selected by members of the Community Advisory Board through nearly 2 years discussions preceding implementation. Across the 2016-2017 academic year (year 0), trained researchers and Head Start staff collected baseline data for all programs. The first group of programs (*n* = 5 programs) began the intervention in fall 2017 (year 1). The second group of programs (*n* = 5 programs) began in fall 2018 (year 2). While the third group of programs (*n* = 6 programs) began the intervention in fall 2019 (year 3) as planned, all intervention activities in all groups were prematurely halted in spring 2020 due to the COVID-19 pandemic (Table 2). As a result, the media campaign and enhanced nutrition support were only implemented for six out of the planned 10 months and no programs implemented PConnect in year 3. The inability to implement PConnect in year 3 meant that parents of children enrolled in group 3 programs never had an opportunity to participate in PConnect and those with children at group 2 and group 1 programs had one (rather than two) and two (rather than three) opportunities to participate, respectively. In addition to the reduction in intervention exposure, the outcome measures were not collected in spring of year 3. Consequently, the investigators elected to evaluate the trial based on data from the first 3 years of the trial (i.e., years 0, 1, 2). This decision was documented in an addendum to the original protocol [54] and a modification of the trial registration. Noteworthy implications of having to end the trial early were that the overall intervention exposure was half of that intended and group 3 programs were only in the control condition; group 1 and 2 programs had periods in the control and intervention conditions.

**Table 2:**
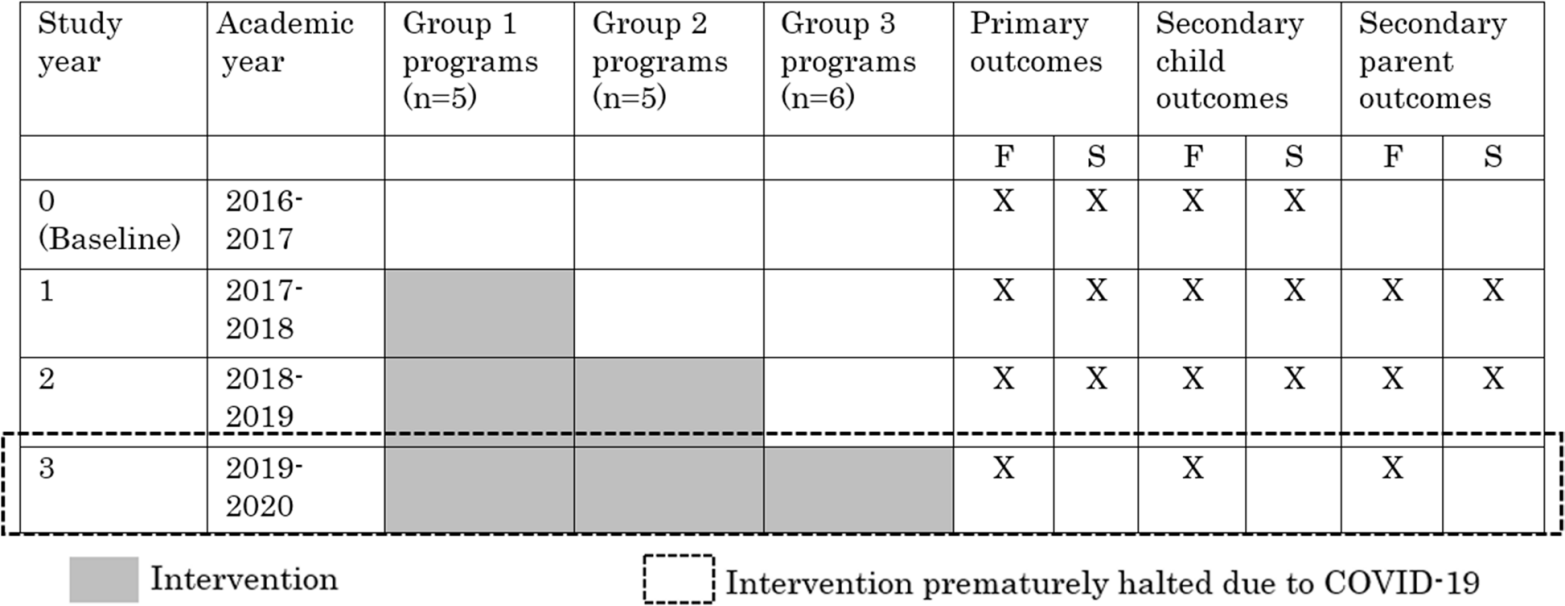
Overview of study timeline

### Participant flow

Throughout each academic year, the media campaign and enhanced nutrition support components of the CHL intervention were integrated into the Head Start’s service provision model at the intervention sites; therefore, in theory, all families enrolled at Head Start programs in the intervention condition were exposed to the intervention. Eligibility criteria for the inclusion of children and parents in the analyses were as follows: children (age 33 months to 5 years, per Head Start eligibility criteria) enrolled in Head Start for the full school year (i.e., enrolled by October 15 and maintained enrollment until at least May 15, with no more than 30 days of leave during the school year) and their parent (or primary caregiver). Families with children who dropped out early from Head Start (i.e., prior to May 15) or those with more than 30 days absent during the school year were not included in the analytic sample, as these individuals were missing critical evaluation data and did not experience the full extent of intervention offered. Between fall 2016 and spring 2019, a single family could be enrolled up to 3 years; if a single family had at least one child enrolled all 3 years, that family would contribute three “family-years” of data. In the figures, tables, and text that follow, we refer to a family-year as a family, a child-year as a child, and a parent-year as a parent, which reflects the long data format.

**Fig. 1:**
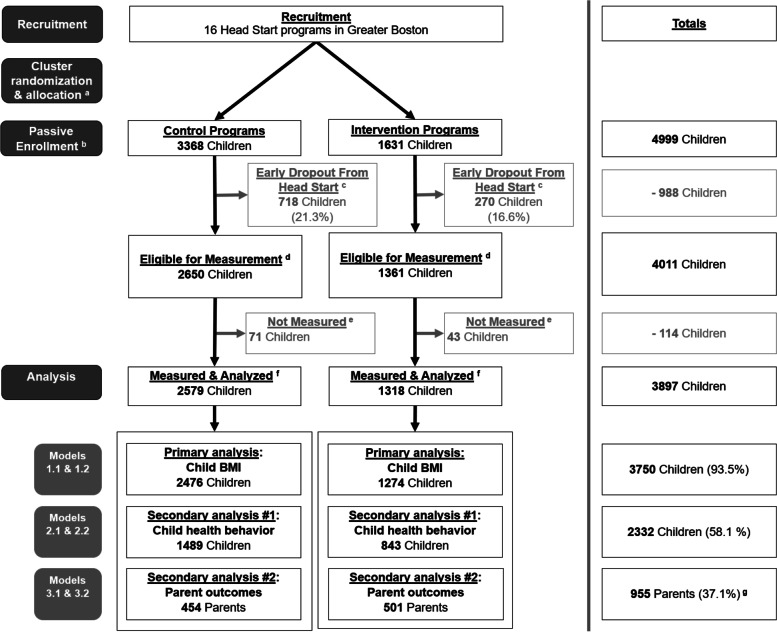
Participant flow: CHL. ^a^ Allocation: Shown is the total number of children enrolled in control and intervention programs, following program-level cluster-randomization at the start of each school year (2016, 2017, 2018). ^b^ Enrollment: Shown is the total number of children enrolled at the start of the combined school years. ^c^ Early dropout from Head Start: Shown is the total number of children who dropped out of Head Start early and did not complete the full school year. ^d^ Eligible for measurement: Shown are the total children eligible for measurement (height, weight, child health behaviors); this includes all children who were enrolled for a full school year during the study period at participating Head Start programs. ^e^ Not measured: Shown is the total number of parent- and child-years lacking sufficient data for inclusion in the analysis. The parent outcomes and child BMI z-score analyses require at least one semester of data within a given school year; the child health behavior analysis requires two semesters of data within a given school year. Those who did not meet these requirements were not included in the analysis. ^f^ Measured & analyzed: Included in the analysis are parent-years with at least one semester of parent outcome measures, and child-years with at least one semester of BMI measures and/or two semesters of child health behavior data. ^g^ 2574 parents were eligible for measurements; only one parent per family unit was invited and eligible to complete the parent survey; note that the number of eligible children exceeds the number of parents eligible for measurement, as only children (no parents) were measured in the baseline school year (2016-2017)

Participant flow is summarized in Fig. 1. Across the 16 Head Start programs recruited for the trial, a total of 4999 children were enrolled at the start of a school year during the study period (fall 2016 - spring 2019). Of these, 988 children dropped out of Head Start early and were excluded from the analyses. For the parent secondary outcomes (i.e., parental empowerment and health-weight parenting practices), 955 parents (37.1% of those eligible) were included in the analyses. Of those enrolled in intervention programs (*n* = 1569), 84 parents enrolled and participated in PConnect, following recruitment via informational flyers and sign-ups at parent meetings [37].

### Data collection

Table 3 summarizes the primary and secondary outcomes, the data sources and data collection methods, the timing of measurement for each outcome, the variables derived for analysis and the associated sample sizes. The specific items utilized are reported elsewhere [37]. All measures were collected during windows representative of “fall” (September-December) and “spring” (April-June), which are referred to hereafter as baseline and follow-up, within each academic year.

**Table 3:** Summary of data sources and collection procedures

Outcomes	Data collection methods	Variables derived for analysis	Sample Size (% of eligible)
**Primary: Child**			
BMIz Modified BMIz	Child height and weight measured in person by trained Head Start health services staff. We extracted the resulting data from administrative records.	BMIz calculated with the Centers of Disease Control and Prevention 20,000 growth charts [55]	3750 children (93.5%)
**Secondary: Child**			
Dietary intake [56, 57] (items = 5) Physical activity [58] (items = 2) Screen time [57] (items = 2) Night sleep duration [59] (items = 2)	Parents surveyed in person by trained Head Start health services staff via enrollment and/or home visit protocols. We extracted the resulting data from administrative records.	Responses dichotomized as meets recommendations (vs. does not): a. Consume vegetables, fruit & water multiple times per day [Note: measured via separate items (*n* = 3)] [60] b. Consume SSBs rarely or never [60] c. Consume juice no more than daily [60] d. Be physically active at least 60 minutes/day [61] e. Spend no more than 60 minutes/day on screen time [62] f, Sleep 10-13 hours per night [63]	2332 children (58.1%)
Weight-related parenting practices [34] (items = 10) Parental empowerment [47] (items = 15)	Parents self-reported survey responses. Trained bilingual research assistants recruited and offered assistance.	Composite score [1–4] calculated by averaging across all items [64–69]; higher scores indicate higher empowerment or healthier parenting practices	955 parents (37.1%)

#### Head start administrative data

Existing data compiled by Head Start during fall and spring of years 0, 1 and 2 (i.e., academic years 2016-2017, 2017-2018 and 2018-2019) for all enrolled children were utilized to operationalize children’s weight status (primary outcome) and health behaviors, including diet, physical activity, sleep, and screen time (child secondary outcomes). Consistent with federal guidelines, Head Start health services and/or nutrition staff (e.g., nutritionists and nutrition case managers) measured child height and weight within 45-90 days of enrollment for every semester enrolled; measures were collected in person during school hours. To enhance data accuracy [70], the CHL study team trained all health services staff in standardized procedures for measuring child height and weight in the fall of each school year (2016-2018). In addition, during the annual enrollment process in early fall, Head Start staff administered a brief survey to families (one parent per family) assessing child health behaviors. Surveys were administered English, Spanish, Chinese, Haitian Creole, Portuguese, Somali, and Arabic. The survey was re-administered in spring to the same parent in each family during mandated home visits. Head Start staff entered the resulting height, weight and health behavior data into each child’s record in the Head Start administrative database.

Families were informed during enrollment each year of the health measures collected and of the potential for de-identified data for all children to be used for quality improvement or research purposes. Passive consent procedures were used for the aforementioned measures; that is, families were given the opportunity to opt out of the assessments prior to measurement. De-identified data for child and family demographics and children’s height, weight and health behaviors were extracted from Head Start administrative records for all families who did not opt out and transferred to CHL’s secure server.

#### New data collected by CHL research team

To augment the data compiled by Head Start, the research team invited parents of children enrolled at participating Head Start programs (one parent per family) to complete a supplemental survey that measured parenting practices and parent empowerment (secondary parent outcomes) along with intervention exposure. Surveys were administered in English, Spanish or Chinese during fall of years 1 (2017) and 2 (2018). Copies of the survey in parents’ preferred language were sent home in children’s schoolbags along with an information sheet and informed consent form. In addition, members of the CHL research team met parents at drop off and pick up to invite them to complete the survey. Parents who completed the survey in fall were invited to complete it again in spring; they received a $10 gift card for each survey completed. Parent survey responses were linked, with parent consent, to demographic and child outcome data (i.e., height, weight, health behaviors) extracted from Head Start records. The institutional review boards of Harvard T.H. Chan School of Public Health and Boston College reviewed and approved study procedures.

### Measures

#### Primary outcomes: child BMIz and modified BMIz

The primary outcomes include change (i.e., spring minus fall score) in child age- and sex-specific BMIz and modified BMIz, which is the most frequently reported outcome in the obesity prevention literature [71]. Though BMIz is the most frequently reported, modified BMIz is also included, as it is more appropriate in longitudinal models [72] and is more sensitive to change among children with high BMIs [73, 74]. For reference, a scatterplot of BMIz vs. modified BMIz measured each fall of the study period among Head Start children included in the analysis (*n* = 3750) (Additional file 1: Supplemental Fig. 1). Child height and weight data were used to calculate BMIz and modified BMIz using the Centers of Disease Control and Prevention (CDC) 20,000 growth charts [55].

#### Secondary outcomes: child health behaviors

Child-level secondary outcomes include dietary intake (vegetables, fruit, sugar-sweetened beverages (SSBs), water, and juice), physical activity, screen time and night time sleep duration. All items (*n* = 11) assessing child secondary outcomes were drawn from validated scales, including Harvard Service Food Frequency Questionnaire [56], the School Physical Activity and Nutrition Survey [57], Burdette’s screener on outdoor play [58], and the extended version of the Brief Infant Sleep Questionnaire (BISQ) [59]. For analysis, responses were converted to a dichotomous indicator, representing whether the child met or did not meet health behavior recommendations (i.e., based on reported dietary intake or duration of time spent in physical activity, screen time or sleep), as defined by the Dietary Guidelines for Americans [60], the Centers for Disease Control and Prevention [61], the American Academy of Pediatrics [62], and the American Academy of Sleep Medicine [63]; the cutoffs for each child health behavior recommendation are described in Table 3.

#### Secondary outcomes: parental empowerment and parenting practices

Parent-level secondary outcomes include parental empowerment (*n* = 15 items) and weight-related parenting practices (*n* = 9 items, scale = 1-4). The survey items measuring each construct were developed and validated by the CHL study team as previously described [34, 37, 47]. The Obesity Parenting for Intervention (OPTION) scale [34] measures three domains of parenting including food, physical activity, and media parenting. The Parental Empowerment through Awareness, Relationships, and Resources (PEARR [47]) scale assesses the development of context-specific critical awareness (i.e., the ability to identify available resources to support children’s health) and the identification of personal relationships useful for child health promotion. For both parental empowerment and parenting practices, a composite score was calculated by averaging across all items (response range: 1-4) to streamline data analysis, as others have done in the past [64–69]. Higher scores indicated higher empowerment or healthier parenting practices.

#### Demographic and socioeconomic variables

Parent- and child-level demographic data extracted from Head Start administrative records included: parent and child age, sex, race, ethnicity; parent level of spoken English proficiency, level of educational attainment, employment status; number of parents in the household and number of children in the home.

#### Process evaluation measures

A comprehensive process evaluation is outlined in a dedicated protocol paper and will be published separately. Accordingly, only brief process information is included here. To document intervention implementation, the total number of brochures distributed, nutrition/wellness staff trained and PConnect sessions implemented were tracked. In addition, as proxy indicators of intervention exposure, parents responded to two groups of questions on the parent survey in spring of years 1 and 2: (1) Since the beginning of this school year, *did you read brochures or flyers about children’s* (a) sugary beverage intake, (b) nutrition, (d) physical activity, (e) screen time and (f) sleep; and (2) Since the beginning of this school year, *did anyone at Head Start speak with you about your child’s* (a) sugary beverage intake, (b) nutrition, (d) physical activity, (e) screen time and (f) sleep. Each health behavior was assessed separately using a yes/no response format.

### Statistical analyses

#### Preliminary analyses

We tabulated the frequencies of demographic and socioeconomic variables, as well as child weight status (as defined by the 2000 CDC Growth Chart for the U.S. [55]) for children eligible for measurement, those included in the analysis (i.e., those for whom we had data and among those in the control vs. intervention arms), and those ineligible for inclusion (due to early dropout from Head Start). This breakdown is presented to assess (1) the extent to which children who were included in the analytic sample were *representative* of all children enrolled in participating Head Start programs (external validity) and (2) the degree of *exchangeability* of participants across trial arms. We reproduced this same table for child- and parent-level secondary analyses. We used linear regression to assess whether missingness was associated with any child- or parent-level outcomes reported.

#### Analysis of primary and secondary outcomes

We conducted analyses in SAS software version 9.4 (Cary, NC) and defined statistical significance as estimates with a *p*-value less than or equal to 0.05. All analyses were planned and undertaken with the oversight from the study statistician (S. Haneuse). For the models examining the primary and secondary outcomes, we ran both unadjusted and adjusted. For adjusted models, covariates were selected a priori to address known confounders documented in the literature (parent race and ethnicity, educational attainment, and household employment status) [75–77]; we also confirmed post hoc that these covariates addressed key observed imbalances in the treatment arms.

For the primary outcomes, we used linear mixed effects regression to estimate mean unadjusted and adjusted differences in BMIz and modified BMIz change per school year from baseline to follow up for the intervention relative to control. Random intercepts accounted for nesting of timepoints (*n* = 2), within children (*n* = 3750), within programs (*n* = 16). As sensitivity analyses, models were re-run by child weight status, as defined by 2000 CDC Growth Charts for the United States [55] (i.e., underweight: < 5th percentile, healthy weight: 5th-85th percentile, overweight: 85th-95th percentile, obesity: ≥ 95th percentile).

For child-level secondary outcomes, we used logistic mixed effects regression to estimate (in odds ratios) the effect of intervention on health behavior recommendations at follow-up (controlling for baseline). Random intercepts accounted for nesting of children (*n* = 2332) within programs (*n* = 16). For parent-level secondary outcomes, we used linear mixed effects regression to estimate mean change in empowerment and parenting among parents exposed to intervention relative to control per year. Random intercepts accounted for nesting of timepoints (*n* = 2) of measurement within parents (*n* = 955), within programs (*n* = 16). As a supplementary analysis, we reproduced the same statistics for parents in intervention programs who chose to participate (post-randomization) in the parenting program (CHL with PConnect; *n* = 55) vs. not (CHL without PConnect; *n* = 440).

#### Sensitivity analyses

As sensitivity analyses, all models were rerun without full year of enrollment as an inclusion criterion. To examine potential heterogeneity in effect by study year, all models were rerun with inclusion of a treatment (CHL) by study interaction. To assess the impact of PConnect dosage, we used linear mixed effects regression to examine differences in parent empowerment and parenting practices by PConnect exposure (e.g., high dose (graduated PConnect), low dose (attended but did not graduate), vs. no dose (exposed to CHL without PConnect).

Process evaluation data were tabulated to verify that intervention activities were implemented as planned and to assess intervention exposure. For the latter, we used chi-square analysis to examine differences (intervention vs. control) in parents’ reports of whether they read brochures or flyers about children’s health behaviors and whether they discussed their child’s health behavior with someone at Head Start. A separate analysis was conducted for each behavior.

## Results

### Sample characteristics

Of all eligible children (*n* = 4011), 3750 (93.5%) were included in the assessment of the primary outcomes (i.e., BMIz and modified BMIz) and 2332 (58.1%) were included for the secondary child outcomes (i.e., child health behaviors related to dietary intake, physical activity, screen time, and sleep). Of the 2574 eligible parents, 955 (37.1%) completed at least one parent survey and were included in the assessment of parent-level secondary outcomes. Consistent with the demographics of families enrolled in Head Start, across all samples, more than 80% of families identified as Non-Hispanic Black/African American or Hispanic, approximately 60% had 1 parent in the household (typically the mother), 25% had less than a high school education and 20% were unemployed.

Demographic and socioeconomic characteristics of those included in the analyses of the primary and secondary outcomes resembled the characteristics of those eligible for inclusion; that is, children and parents included in the analyses were representative of all families enrolled in the participating Head Start programs (Table 4 for primary outcomes, Additional file 1: Supplemental Table 1 for child-level secondary outcomes, and Additional file 1: Supplemental Table 2 for parent-level secondary outcomes). Furthermore, for the primary outcomes, the characteristics of children eligible vs. ineligible for inclusion largely resembled one another with two exceptions: children deemed ineligible were more likely to be older (age 4-5) and have parents who identified as unemployed (Table 4).

**Table 4:** Characteristics of Greater Boston Head Start children included in the primary analysis and their parents by eligibility, inclusion, and treatment arm, 2016-2019 (*n* = 4011)

	Eligible ^a^	Included in Final Sample ^b^			Ineligible ^a^
	All (*n* = 4011)	All (*n* = 3750)	Control (*n* = 2476)	Intervention (*n* = 1274)	All (*n* = 988)
	%	%	%	%	%
Child age (months) ^c^					
2 years old	11.2	11.2	11.0	11.4	8.1
3 years old	53.8	53.8	53.9	53.5	40.2
4 years old	35.0	35.1	35.1	35.1	51.7
Child sex					
Male	49.0	49.1	50.2	49.0	50.5
Female	51.0	50.9	49.8	51.0	49.5
Parent race/ethnicity					
NH Asian	9.6	9.4	7.2	13.9	7.2
NH Black/AA	34.1	34.1	41.2	20.3	35.0
Hispanic/Latino	40.9	41.2	37.0	49.3	43.0
NH Other ^d^	1.9	1.9	1.8	2.1	2.5
NH White	8.2	8.2	6.2	12.2	6.3
Missing	5.3	5.2	6.6	2.2	6.0
Parent level of education					
< High school	24.2	24.3	22.7	27.3	21.3
High school	38.6	38.9	39.1	38.5	37.7
> High school	33.5	33.2	34.4	30.8	38.3
Missing	3.7	3.6	3.8	3.4	2.8
Parent employment					
Unemployed	25.7	25.6	28.6	19.7	34.4
Employed	22.8	22.9	18.9	30.7	23.2
Other ^e^	48.9	48.9	49.7	47.4	40.7
Missing	2.6	2.6	2.8	2.2	1.7
No. of parents in home					
1 (vs. 2)	63.1	63.2	67.4	55.2	70.7
Baseline weight status ^*f*^					
Underweight	3.0	3.3	2.9	3.9	1.5
Healthy weight	54.0	57.8	58.3	56.8	31.5
Overweight	14.6	15.6	16.4	14.1	6.8
Obese	17.1	18.2	18.3	18.2	8.9
Missing	11.3	5.1	4.2	7.0	51.3

Shown is the percent

^a^ Children eligible for survey completion must be enrolled at a participating Head Start program for a full school year (2016-2019). Those who dropped out of Head Start early were considered ineligible for inclusion

^b^ Those included in the analysis have at least one semester of height and weight data available (i.e., baseline and follow-up measures of a given school year)

^c^ Age is that on September 1 of the school year enrolled; calculated from date of birth; 2-year-olds are aged 29-35 months, 3-year-olds are aged 36-47 months, and 4-year-olds are aged 48-59 months

^d^ “Other” category includes individuals who identified as biracial/multiracial, as well as Native American, Alaska Native, Native Hawaiian or Pacific Islander

^e^ “Other” includes those who reported that they are in a job training program, school full-time or part-time, employed full-time or part-time, in a paying job, “not applicable,” or “other”

Responses.

^f^ As defined by the 2000 CDC Growth Chart for the U.S. based on child height and weight measurements

We also examined the comparability or exchangeability of the children and parents in intervention versus control conditions (Table 4 for primary analyses; Additional file 1: Supplemental Table 1 for secondary child-level analyses; and Additional file 1: Supplemental Table 2 for supplemental parent-level analyses). Child gender and age were comparable for the control (vs. intervention); across both conditions, approximately 50% of children were female (49.8 vs. 51.0%) and the vast majority were three- (53.9% vs 53.6%) or four- (35.1% both arms) years-old at the start of the school year. Aside from these few similarities, notable differences were observed. Proportionately more families in the control (vs intervention) identified as Black (41.2% vs. 20.3%), reported they were unemployed (28.6% vs. 19.7%), and resided in a single-parent household (67.4% vs. 55.2%). Conversely, proportionately fewer families in the control (vs. intervention) were Hispanic (37.0% vs. 49.3%) and had less than a high school education (22.7% vs 27.3%). A similar pattern of differences was identified for control vs. intervention for the parent outcomes (Additional file 1: Supplemental Table 2). In the adjusted models, we specifically accounted for parent race/ethnicity, education, and employment status.

### Missing data

We present the frequency of missingness for all measured covariates (Table 4). Of the 3750 children for whom we had height and weight data available for the primary analysis, 3467 (92.5%) had complete data for covariates and 283 children (7.5%) were missing data for at least one covariate in the adjusted model. Of the 2332 children for whom we had secondary child health behavior data, 2165 (92.8%) had complete data and 167 were missing at least one covariate included in the adjusted model. Of the 2574 parents eligible for measurement, 955 (37.1%) responded to the parent survey for at least one semester of the study period; of these, 912 (95.5%) had complete data and 43 were missing at least one covariate included in the adjusted model (4.5%). Of all the outcomes reported, missingness in key parent-level confounders were only associated with the odds of meeting water intake recommendations at follow-up, and the magnitude of association was small, the confidence intervals wide, and the significance marginal (OR = 0.73, 95% CI = 0.54, 0.99). Due to the relatively limited missingness and the lack of evidence of association between outcome reporting and missingness, all analyses are run as likelihood-based complete case analyses [78–80], which yield valid results under the missing at random assumption [81]. Given the relatively limited missingness for the covariates and lack of associations with the outcome variables, as reported earlier, we conducted all subsequent analyses as likelihood-based complete case analyses.

### Primary outcomes

The mean (SD) BMIz and modified BMIz scores at baseline, and change from baseline to follow-up, by trial arm along with results from the unadjusted and adjusted models are presented in Table 5. Children enrolled at control programs demonstrated a higher unadjusted mean (SD) BMIz and modified BMIz at baseline than those enrolled at intervention programs (respectively, control vs. intervention; 0.64 (1.20) vs. 0.58 (1.26); 0.63 (1.40) vs. 0.57 (1.42)). Contrary to our a priori hypotheses, children enrolled in intervention programs demonstrated a small, yet statistically significant, mean increase in BMIz and modified BMIz per year beyond that experienced by children enrolled at control programs, after controlling for key confounders (Model 1.2; *b* = 0.06, 95% CI = 0.02, 0.10; *b* = 0.07, 95% CI = 0.03, 0.12). When analyses were rerun by child weight status, increases in modified BMIz were significantly greater in the intervention vs. control group among those of healthy weight (*b* = 0.05, 95% CI = 0.008, 0.10) and those with obesity (*b* = 0.19, 95% CI = 0.08, 0.29; Additional file 1: Supplemental Table 3).

**Table 5:** Primary outcomes: Estimated mean change in BMIz and modified BMIz scores from baseline to follow-up among Greater Boston Head Start children (*n* = 3750)

	Mean (SD) values by timepoint		Mean (SD) values by timepoint		Estimated change in outcome (Intervention vs. control)	
	Control (*n* = 2476)		Intervention (*n* = 1274)		Model 1.1	Model 1.2
	Baseline	Change	Baseline	Change	Unadjusted ^a, b^	Fully Adjusted ^a,^^c^
BMIz	0.64 (1.20)	−0.01 (0.42)	0.58 (1.26)	0.04 (0.44)	0.06 (0.02, 0.09) **	0.06 (0.02, 0.10) **
Modified BMIz	0.63 (1.40)	−0.02 (0.45)	0.57 (1.42)	0.05 (0.46)	0.07 (0.03, 0.10) ***	0.07 (0.03, 0.12) **

^a^ Stars indicate significance *p ≤ 0.05, **p ≤ 0.01, ***p ≤ 0.001. Baseline = fall; follow-up = spring; change = follow-up – baseline

^b^ Results from an unadjusted linear mixed effects regression model; shown is the mean unadjusted change (95% confidence interval) in outcomes for children exposed to intervention relative to control

^c^ Results from a linear mixed effects regression model, adjusting for parent race and ethnicity, educational attainment, and household employment status; shown is the mean adjusted change (95% confidence interval) in outcomes for children exposed to intervention relative to control

### Secondary outcomes: children

The frequency of children meeting health behavior recommendations by timepoint and intervention status along with the results of the unadjusted and adjusted models are reported in Table 6. Across both study arms, a minority of children met recommendations related to daily fruit and vegetable intake and screen time at baseline and follow-up, whereas a majority met recommendations related to daily beverage intake, time spent in physical activity, and night sleep duration. After adjusting for covariates, the odds of meeting recommendations for SSB intake (i.e., rarely consume; *b* = 1.5, 95% CI = 1.1, 2.1), water intake (i.e., consume multiple times per day; *b* = 1.6, 95% CI = 1.2, 2.3), and screen time (i.e., engage in screen time ≤ 1 hour per day; *b* = 1.4, 95% CI = 1.0, 1.8) were on average significantly higher among children enrolled at intervention vs. control programs (*b* = 1.5, 95% CI = 1.1, 2.1; *b* = 1.6, 95% CI = 1.2, 2.3; *b* = 1.1, 95% CI = 0.68, 1.8; *b* = 1.4, 95% CI = 1.0, 1.8) (Model 2.2, Table 6). No significant intervention effects were identified for vegetable intake, fruit intake, juice intake, physical activity, or night sleep duration in unadjusted (Model 2.1) or adjusted models (Model 2.2).

**Table 6:** Secondary outcomes (children): Estimated odds of meeting health behavior recommendations at follow-up among Greater Boston Head Start children (*n* = 2332)

	Percent meeting recommendations by timepoint		Percent meeting recommendations by timepoint		Estimated odds of meeting recommendations at follow-up (Intervention vs. control)	
	Control (*n* = 1489)		CHL (*n* = 843)		Model 2.1	Model 2.2
	Baseline	Follow-up	Baseline	Follow-up	Unadjusted ^a,b^	Fully Adjusted ^a,c^
Vegetable	13.8	16.6	17.3	16.0	0.90 (0.68, 1.2)	0.94 (0.66, 1.3)
Fruit	25.5	29.0	27.9	28.8	0.84 (0.66,1.1)	1.0 (0.75, 1.4)
SSB	72.1	73.5	73.2	79.6	1.5 (1.1, 1.9) **	1.5 (1.1, 2.1) *
Water	59.8	61.6	70.5	73.0	1.5 (1.2, 2.0) ***	1.6 (1.2, 2.3) **
Juice	87.0	90.3	88.1	90.9	1.1 (0.77, 1.6)	0.95 (0.60, 1.5)
Physical Activity	74.7	82.3	83.8	84.6	1.1 (0.73, 1.5)	1.1 (0.68, 1.8)
Screen time	15.0	15.7	17.8	21.1	1.4 (1.1, 1.7) **	1.4 (1.0, 1.8) *
Night sleep duration	78.0	80.4	79.7	78.1	0.91 (0.70, 1.2)	0.91 (0.69, 1.2)

^a^ Stars indicate significance *p ≤ 0.05, **p ≤ 0.01, ***p ≤ 0.001

^b^ Results from an unadjusted linear mixed effects regression model; shown is the mean unadjusted change (95% confidence interval) in outcomes for children exposed to intervention relative to control

^c^ Results from a linear mixed effects regression model, adjusting for parent race and ethnicity, educational attainment, and household employment status; shown is the mean adjusted change (95% confidence interval) in outcomes for children exposed to intervention relative to control

### Secondary outcomes: parents

The mean (SD), and absolute mean change, in parental empowerment and parenting practice scores by intervention status and results of the unadjusted and adjusted models are presented in Table 7. Mean (SD) empowerment and parenting practice scores were comparable at baseline for parents from intervention vs. control programs (3.2 (0.42) vs. 3.0 (0.40)). No significant differences in the relative changes in parent empowerment nor parenting practices, were observed for parents in intervention vs. control programs (Model 3.1 and 3.2).

**Table 7:** Secondary outcomes (parents). Mean parental empowerment and parenting scores by timepoint, absolute change across semesters, and modeled relative mean change among parents exposed to intervention vs. control

	Mean (SD) values by timepoint		Mean (SD) values by timepoint		Estimated change in outcome (Intervention vs. control)	
	Control (*n* = 454)		Intervention (*n* = 501)		Model 3.1 ^a,b^	Model 3.2 ^a,c^
	Baseline	Change	Baseline	Change	Unadjusted	Adjusted
Empowerment	3.2 (0.41)	0.02 (0.50)	3.2 (0.40)	0.05 (0.40)	0.04 (−0.02, 0.11)	0.05 (−0.02, 0.11)
Parenting	3.0 (0.40)	−0.02 (0.37)	3.0 (0.40)	0.05 (0.36)	0.05 (−0.003, 0.11)	0.06 (0.00, 0.12)

^a^ Stars indicate significance *p ≤ 0.05, **p ≤ 0.01, ***p ≤ 0.001

^b^ Results from an unadjusted linear mixed effects regression model; shown is the mean unadjusted change (95% confidence interval) in outcomes for parents exposed to intervention relative to control

^c^ Results from a linear mixed effects regression model, adjusting for parent race and ethnicity, educational attainment, and household employment status; shown is the mean adjusted change (95% confidence interval) in outcomes for parents exposed to intervention relative to control

In a supplemental analysis, we assessed relative differences in empowerment and parenting practices for parents in intervention programs who enrolled in PConnect vs. did not. During the 2 years that PConnect was offered, 84 parents participated in at least one PConnect session; of these, 56 completed at least one survey measuring parent outcomes (see Additional file 1: Supplemental Fig. 2 for participant flow). Further, those who chose to enroll in PConnect resembled those enrolled in the larger intervention group, with a couple of exceptions: PConnect parents were more likely to identify as Hispanic/Latino and report being employed (Additional file 1: Supplemental Table 2). However, it should be noted that there is still a possibility of selection bias, as those who participated elected to join the program.

As shown in Table 8, mean (SD) parental empowerment and parenting practice scores were similar for those exposed to the intervention without PConnect vs. those who chose to enroll in PConnect (respectively; 3.2 (0.39); 3.0 (0.47)). Unadjusted mean (SD) increases in parental empowerment and parenting practices were several times greater among those who chose to participate in PConnect vs. not ((empowerment: 0.15 (0.36) vs. 0.03 (0.41); parenting: 0.14 (0.34) vs. 0.03 (0.37)). After adjustment for covariates, the difference was statistically significant for empowerment (*b* = 0.17; 95% CI = 0.04, 0.31) but not for parenting practices (*b* = 0.04; 95% CI = -0.09, 0.18; Model 4.2).

**Table 8:** Supplemental analyses (Parents). Mean parental empowerment and parenting scores by timepoint, absolute change across semesters, and modeled relative mean change among parents exposed to intervention with PConnect vs. without PConnect

	Mean (SD) values by timepoint		Mean (SD) values by timepoint		Estimated change in outcome (Intervention with PConnect vs. without)	
	Intervention without PConnect (*n* = 440)		Intervention with PConnect (*n* = 55)		Model 4.1 ^a,b^	Model 4.2 ^a,c^
	Baseline	Change	Baseline	Change	Unadjusted	Adjusted
Empowerment	3.2 (0.39)	0.03 (0.41)	3.2 (0.47)	0.15 (0.36)	0.17 (0.03, 0.31) **	0.17 (0.04, 0.31) **
Parenting	3.0 (0.40)	0.03 (0.37)	3.0 (0.38)	0.14 (0.34)	0.05 (−0.08, 0.18)	0.04 (−0.09, 0.18)

^a^ Stars indicate significance *p ≤ 0.05, **p ≤ 0.01, ***p ≤ 0.001

^b^ Results from an unadjusted linear mixed effects regression model; shown is the mean unadjusted change (95% confidence interval) in outcomes for parents exposed to intervention with PConnect vs. without PConnect

^c^ Results from a linear mixed effects regression model, adjusting for parent race and ethnicity, educational attainment, and household employment status; shown is the mean adjusted change (95% confidence interval) in outcomes for parents exposed to intervention with PConnect vs. without PConnect

### Sensitivity analyses

When models were rerun including children enrolled in Head Start for less than a year, the results were nearly identical to those for the primary models which specified a full year of enrollment data as an inclusion criterion (data not shown). For the majority of models, the effect of the intervention (CHL) exposure was not found to statistically significantly differ by study year; the two exceptions were meeting recommendations related to physical activity and water intake. Specifically, the estimated relative odds of meeting physical activity and water intake recommendations (among those exposed to intervention vs. control) were lower in study year 1 vs. 2 (*b* = − 1.42; 95% CI = -2.11, − 0.72; *b* = − 0.73, 95% CI = -1.24, − 0.23, respectively; Additional file 1: Supplemental Table 5). When comparing findings among parents exposed to high and low dose PConnect (e.g., graduated and not) vs. CHL without PConnect, we found that those exposed to low dose PConnect demonstrated insignificant relative differences in empowerment and parenting practices, whereas, those exposed to high dose PConnect demonstrated statistically significantly higher relative increases in empowerment (*b* = 0.28; 95% CI = 0.11, 0.44), as hypothesized (Additional file 1: Supplemental Table 6).

### Process evaluation

Process evaluation indicators measured intervention implementation and exposure. In year 1 of the intervention, when five out of 16 programs were in the intervention group, at least 1400 flyers were distributed to families (to the 542 enrolled families), 56 Head Start nutrition staff and teachers were trained (71-100% of eligible staff) and 36 PConnect sessions were implemented. In year 2, when 10 out of 16 programs were in the intervention, at least 5000 flyers were distributed (to the 765 enrolled families), 132 Head Start staff were trained (47-100% of eligible staff) and a total of 72 PConnect sessions were implemented.

Parents’ reported exposure to nutrition support services and health promotion materials, activities consistent with CHL, were used as proxy indicators for intervention exposure. While we anticipated that parents would report both of these activities in the intervention and control conditions (given Head Start standard practice), we expected they would be reported at significantly higher rates in the intervention. Across all child health behaviors (i.e., nutrition, physical activity, screen time, sleep, and sugary drink intake), a significantly greater proportion of parents in the intervention vs. control reported engaging with nutrition services and exposure to relevant printed materials (*p*-value ≤0.01 for all comparisons; Additional file 1: Supplemental Table 4). Across all behaviors, an average of 59.7% of intervention vs. 48.3% of control parents recalled engaging with nutrition support services; 78.5% of intervention vs. 63.2% of control parents recalled reading a related brochure or flyer.

## Discussion

Unexpectedly, we observed a small, statistically significant relative increase in child BMIz and modified BMIz among those enrolled in the intervention (vs. control). However, aligned with a priori hypotheses, we also observed significantly higher odds of children meeting three health behavior recommendations at follow-up, specifically in relation to SSB intake, water intake, and screen time; no significant differences in vegetable, fruit, and juice intake, nor time spent in physical activity and night sleep were observed. Additionally, parents enrolled in the high-intensity PConnect program exhibited significant increases in parental empowerment, relative to those who did not participate in PConnect, though no difference was observed for those exposed to CHL overall (vs. control). These results align well with results form a systematic review of obesity interventions in ECE settings [16]; high intensity parent interventions that were interactive and directly and repeatedly engage parents demonstrated efficacy, whereas low intensity interventions with passive, low-burden intervention components, such as a media campaign and supplemental nutrition support, exhibited low impact on child outcomes.

Although the relative differences in BMIz and modified BMIz are statistically significant (respectively: *b* = 0.06 BMIz units and *b* = 0.07 modified BMIz units), the sizes of these differences are approximately one fourth the magnitude deemed *clinically important* (0.20-0.25 BMIz units) by US Preventative Services Task Force (2017) [82] based on a systematic evidence review [83]. Contrary to clinical significance, the *statistical* significance we observed is likely a product of large sample size [84]. This study draws on nearly 3000 child years of data; few other comparable obesity prevention interventions reach a sample size of 1000 children [85]. Though few prior studies have reported a significant relative increase in BMIz among the intervention vs. control groups, many studies have reported results similar to ours in magnitude and direction (i.e., opposite from expected), but which were not statistically significant. A fundamental difference between our study and these prior studies is sample size. Our sample size is several folds larger than most other studies [86]. The larger effect size observed among children with obesity vs. healthy weight (*b* = 0.19, 95% CI = 0.08, 0.29; *b* = 0.05, 95% CI = 0.008, 0.10), however, may be of concern and warrants further research.

Though unexpected, there are a few possible reasons we observed a small significant increase in BMIz and modified BMIz among the intervention vs. control group. First, the control and intervention groups demonstrated limited exchangeability because randomization was conducted at the program- (vs. child-) level. With the number of programs being relatively small (*n* = 16), achieving perfect balance was unlikely, no matter the random allocation. Furthermore, as previously noted, intervention exposure was half that intended (due to COVID-19-related early study termination); this reduced the duration that families were exposed to the intervention materials and limited Head Start’s ability to optimize implementation over time. It also resulted in a lack of exchangeability across control vs. intervention samples; while every participating Head Start program was meant to transition from control to intervention exposure during the study period, early study termination prevented approximately one third of the programs from ever receiving the intervention and effectively reduced the intervention sample size by half. As a result, the programs assigned to the intervention arm served different communities of families (with unique needs, resources, and environmental contexts) than those assigned to the control arm. While we adjusted for key measured confounders to address this, there may have been differences we did not account for that may explain the results.

At follow-up, the positive impacts observed on relative odds of meeting some child health behavior recommendations (i.e., SSB intake, water intake, and screen time), but not others (i.e., fruit, vegetable, or juice intake; physical activity; and night sleep duration), align with findings from similar lifestyle-based intervention programs in early childhood [18, 87–91]. While most other ECE-based and family-based obesity prevention programs were designed to target one or two specific outcomes [16, 92, 93], the current study adopted a much broader scope, targeting eight child behavior outcomes. The broader scope of this study may explain the mixed results if parents focused on a subset of behaviors, rather than all simultaneously. Additionally, unlike most other programs targeting behavior change in preschoolers, this study did not engage directly with preschoolers [16], but instead targeted parents exclusively. It is possible that parent engagement alone is insufficient. Future iterations of CHL could enhance behavior change outcomes through adaptation of classroom curriculum, activities and/or environment [94] to engage with preschoolers directly, as previous studies have done [95–97]. Further, it is possible that behavior changes observed were limited due to low-level parent engagement with the resources offered [16, 88]; greater emphasis on interactive activities for parents may be warranted.

Children in the intervention exhibited 40-60% higher odds of meeting recommendations for SSB, water and screen time. This relative increase in odds (~ 50%) may be meaningful, as any reduction in sugary beverage consumption may help alleviate risk of, for example, unhealthy weight gain, insulin resistance, and dental caries [98–100]. Increased odds of meeting screen time recommendations are promising, as the literature supports a link between greater sedentary screen time and heightened risk of obesity in early childhood [101, 102]. This observed impact is especially hopeful given the understanding that such a striking minority of children currently meet recommendations [103]. It should be noted that the behaviors for which we observed a measurable impact were those which required restriction of behavior (e.g., reducing sugar-sweetened beverage consumption or decreasing screen time) or an increase in consumption of a nearly free beverage (e.g., water). These behaviors introduce fewer barriers to change than those which require the uptake of something new, which may cost time, money, or scheduling flexibility (e.g., purchase of fruit and vegetables, time required for a sleep routine, space for physical activity) [104, 105].

Finally, we observed a significant, relative increase in parental empowerment among parents in the intervention condition who enrolled in PConnect versus those who did not. The observed relative increase of 0.17 empowerment units (scale: 1-4) translates to a caregiver with PConnect (vs. without) rating higher agreement with roughly two to three empowerment questions at follow-up. For example, a caregiver may report higher agreement with statements, related to the prompt “If I have concerns about my child’s health …” : I know I can get my family to help”, “I ask friends and family for health or advice”, and “I use the programs, services, and other resources in my community to help my child.” Though an abundance of literature cites empowerment as the foundation for intervention development, the measurement literature on empowerment as an outcome in health promotion studies remains limited [106–110]. Of the literature that does exist, most is qualitative [111] and of the quantitative studies published, measures are strikingly diverse [112], thereby hindering the interpretation of findings against others in the field. However, the observed relative increase in this paper may deserve attention, as any increase in the empowerment domains of competency, efficacy, or action are likely beneficial to healthy child growth and development [87, 113–116]. While we observed positive changes in empowerment among the most engaged parents as well as higher odds of meeting health behavior recommendations at baseline among children exposed to the intervention, we did not observe any meaningful subsequent changes in child BMIz or modified BMIz.

Several limitations should be taken into consideration when considering this study’s results. First, it is possible that the intervention dose was too low for the majority of families, particularly those who did not participate in PConnect. While our process evaluation suggests that intervention activities were implemented as planned and that parents in the intervention were more likely to report the receipt of nutrition support and educational materials likely to be exposed to nutrition support, CHL was only implemented for half of the intended time across all programs as a result of the premature conclusion of the study. Coupled with this, rapid turnover of Head Start families precluded our ability to examine change over multiple years for the five programs that implemented CHL over 2 years. An academic school year may not be long enough to observe the translation of parent-level changes (in behavior, cognition, and beliefs) into meaningful changes in child anthropometries.

The use of self-report behavior measures could also be considered a limitation, as self-report methods may introduce measurement error or conscious bias [117] or social desirability bias [118, 119]. However, studies which rely on the alternative of trained observation are often smaller in scale and less diverse [120–122]. Additionally, a number of the survey measures used were validated with children of a slightly different age than our sample; for example, the Brief Infant Sleep Questionnaire has been validated with samples up to 30 months [59, 123] and the diet items were adapted from two measures validated among school-aged children [56, 57]. The validity of the diet items may also be limited by the fact that we included only 5 items of a longer survey. Additionally, we were limited in that we did not have data on parent BMI and could not account for it as a potential confounder. Finally, our operationalization of race/ethnicity as a five-category variable is an oversimplified representation of the social construct of race. We chose to include race/ethnicity as a covariate in our adjusted models, as a means of addressing confounding introduced by race as a social construct, which is known to impact individual-level obesity risk [124, 125] and community-level differences in nutrition and health resource access [124, 125].

In terms of strengths, our use of data compiled by Head Start to operationalize the child outcomes allowed both the diversity and size of our sample to exceed those of most other school-based studies in the field [126, 127]. While most other studies report on results of less than 1000 children [126, 127], for example, we report on nearly double that, representing over 90% of children enrolled at participating programs, as well as nearly 1000 parents (representing > 30% of those eligible). The use of administrative data, and thereby passive enrollment procedures, also helped prevent measurement bias in the assessment of BMI, which is a known issue for studies requiring participants to opt-in [128]. Relatedly, our sample’s representativeness, in comparison to all families enrolled at participating Head Start sites, supports the generalizability of our findings beyond our sample to the larger national Head Start population, particularly sites in urban contexts. Further, the self-report items used in this study were drawn from scales validated for use among diverse families (i.e., the Comprehensive Feeding Practices Questionnaire [129–131], the Activity Support Scale for Multiple Groups [132, 133], the Sleep Parenting Scale for Infants [134], and the Family Empowerment Scale [66, 68, 135, 136]).

## Conclusion

This study offers important implications for on-the-ground research and practice. Grounded in CBPR, CHL serves as a model for community-led measure selection and implementation on a large scale. Study results suggest intervention exposure was associated with statistically but not clinically significant increases in BMIz and increased odds of meeting recommendations for three of eight child behaviors; premature trial suspension may explain mixed results. With the trial prematurely suspended due to the COVID-19 pandemic, we were unable to observe longer-term changes in parenting practices and child behaviors which may have followed these empowerment increases. However, this intervention offers a feasible model for future researchers to build on for the largescale monitoring and modification of parent and child health behavior and weight status in the early childcare education context.

## Supplementary Information

**Additional file 1.**

**Additional file 2.**

**Additional file 3.**

## Data Availability

The datasets used and/or analyzed during the current study are available from the corresponding author on reasonable request.

## Abbreviations

CHL: Communities for Healthy Living
ECE: Early Care and Education
CBPR: Community-Based Participatory Research
CAB: Community Advisory Board
PConnect: Parents Connect for Healthy Living
BMIz: Body Mass Index z-score
CDC: Centers of Disease Control and Prevention
SSBs: sugar-sweetened beverages
(OPTION) scale: Obesity Parenting for Intervention
PEARR: Parental Empowerment through Awareness, Relationships, and Resources
