# Supplementary material for: Evaluation of a cluster-randomized controlled trial: Communities for Healthy Living, family-centered obesity prevention program for Head Start parents and children
Source: Int J Behav Nutr Phys Act. 2023 Jan 11;20:4. doi: 10.1186/s12966-022-01400-2 (PMC9832428; doi:10.1186/s12966-022-01400-2)
Supplement: Supplementary file 1 — Additional file 1. [file 12966_2022_1400_MOESM1_ESM.docx]

## **Supplemental Table 1. Characteristics of Head Start children included in the secondary analysis on child health behaviors and their parents by eligibility, inclusion, and treatment arm, 2016-2019.**

|  |  | **Eligible ^a^** |  | **Included in Final Sample ^b^** | | |
| --- | --- | --- | --- | --- | --- | --- |
|  |  | **All** (n=4011) |  | **All** (n=2332) | **Control** (n=1489) | **Intervention** (n=843) |
|  |  | % |  | % | % | % |
| Child age (months) **^c^** | |  |  |  |  |  |
|  | 2 years old | 11.2 |  | 12.0 | 12.3 | 11.4 |
|  | 3 years old | 53.8 |  | 55.6 | 56.6 | 53.9 |
|  | 4 years old | 35.0 |  | 32.4 | 31.1 | 34.8 |
| Child sex | |  |  |  |  |  |
|  | Male | 49.0 |  | 49.1 | 50.2 | 47.3 |
|  | Female | 51.0 |  | 50.9 | 49.8 | 52.7 |
| Child race & ethnicity | |  |  |  |  |  |
|  | NH Asian | 10.1 |  | 12.4 | 9.1 | 18.3 |
|  | NH Black/AA | 34.9 |  | 32.3 | 40.4 | 17.9 |
|  | Hispanic/Latino | 43.1 |  | 41.8 | 39.4 | 46.0 |
|  | NH Other **^d^** | 4.3 |  | 5.0 | 4.4 | 6.2 |
|  | NH White | 6.2 |  | 6.8 | 4.4 | 11.0 |
|  | Missing | 1.5 |  | 1.7 | 2.3 | 0.59 |
| Parent age (years) **^c^** | | 33.8 |  | 34.1 | 33.9 | 34.4 |
| Parent sex | |  |  |  |  |  |
|  | Male | 3.4 |  | 3.9 | 4.7 | 2.4 |
|  | Female | 95.4 |  | 94.7 | 94.1 | 95.9 |
|  | Missing | 1.3 |  | 1.4 | 1.2 | 1.8 |
| Parent race/ethnicity | |  |  |  |  |  |
|  | NH Asian | 9.6 |  | 11.8 | 8.5 | 17.7 |
|  | NH Black/AA | 34.1 |  | 32.0 | 40.2 | 17.6 |
|  | Hispanic/Latino | 40.9 |  | 40.1 | 37.3 | 45.2 |
|  | NH Other **^d^** | 1.9 |  | 2.4 | 2.3 | 2.5 |
|  | NH White | 8.2 |  | 9.1 | 6.0 | 14.6 |
|  | Missing | 5.2 |  | 4.6 | 5.7 | 2.5 |
| Parent spoken English proficiency | |  |  |  |  |  |
|  | Not at all or not well | 29.9 |  | 33.2 | 27.8 | 42.8 |
|  | Well or very well | 63.4 |  | 59.4 | 64.6 | 50.2 |
|  | Missing | 6.7 |  | 7.4 | 7.6 | 7.0 |
| Parent level of education attained | |  |  |  |  |  |
|  | < High school | 24.2 |  | 25.8 | 24.2 | 28.7 |
|  | High school | 38.6 |  | 38.7 | 38.8 | 38.6 |
|  | > High school | 33.5 |  | 31.4 | 32.6 | 29.3 |
|  | Missing | 3.6 |  | 4.0 | 4.4 | 3.4 |
| Parent employment status | |  |  |  |  |  |
|  | Unemployed | 25.7 |  | 27.6 | 31.4 | 20.9 |
|  | Employed | 22.8 |  | 22.6 | 16.9 | 32.7 |
|  | Other | 48.9 |  | 46.8 | 48.4 | 43.9 |
|  | Missing | 2.6 |  | 3.0 | 3.4 | 2.5 |
| Number of parents in home | |  |  |  |  |  |
|  | Single parent | 63.1 |  | 57.6 | 62.8 | 48.4 |
|  | Two parents | 36.9 |  | 42.4 | 37.2 | 51.6 |
| Number of children in home | |  |  |  |  |  |
|  | 1 | 38.4 |  | 36.7 | 36.6 | 36.9 |
|  | 2 | 38.3 |  | 39.8 | 38.7 | 41.8 |
|  | 3+ | 23.1 |  | 23.3 | 24.6 | 21.1 |
|  | Missing | 0.17 |  | 0.17 | 0.13 | 0.24 |

*Shown is the percent.*

***^a^*** *Children eligible for survey completion must be enrolled at a participating Head Start program for a full school year (2016-2019).*

***^b^*** *Those included in the analysis have at least one semester of height and weight data available (i.e., fall and/or spring measures of a given schoolyear).*

***^c^*** *Age is that in years on September 1 of the school year enrolled; two-year-old aged 29-35 months, 3-year-old aged 36-47 months, and 4-year-old aged 48-59 months.*

***^d^*** *“Other” category includes individuals who identified as biracial/multiracial, as well as American Indian, Alaska Native, Native Hawaiian or Pacific Islander.*

## **Supplemental Figure 1. Scatterplot of BMIz vs. modified BMIz measured in fall of the study period among children enrolled in Head Start (n=3750).**

***
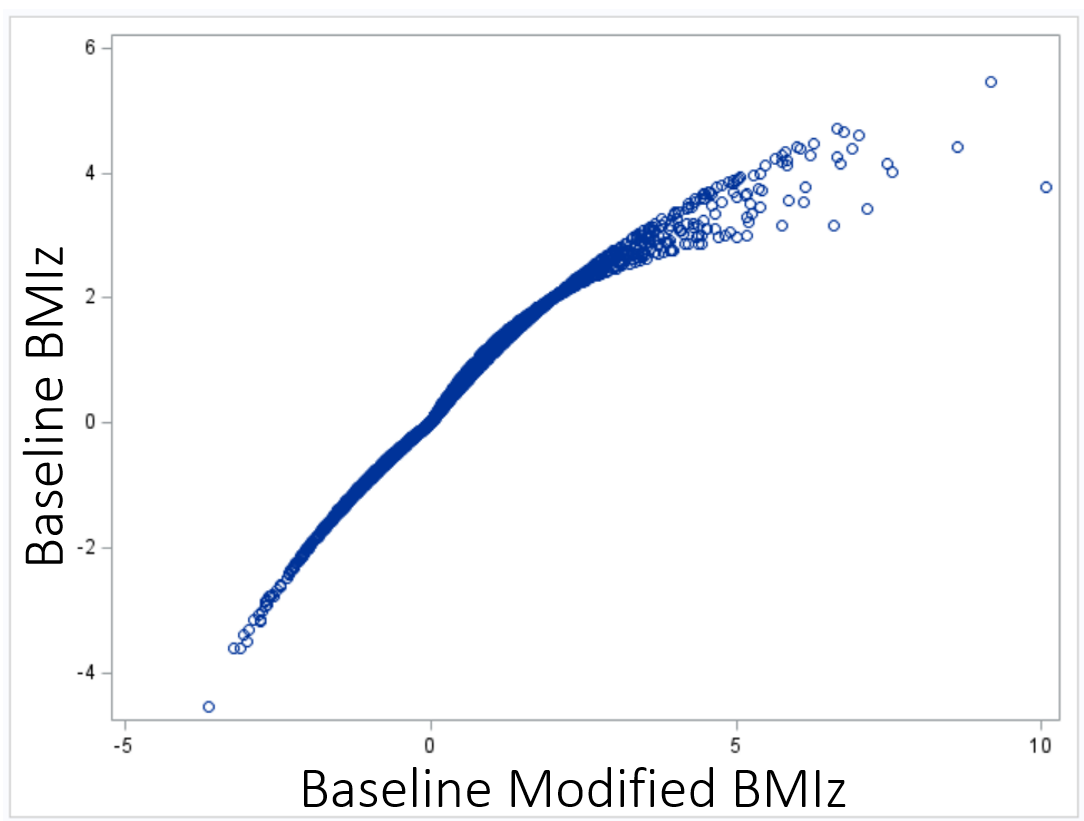
***

**
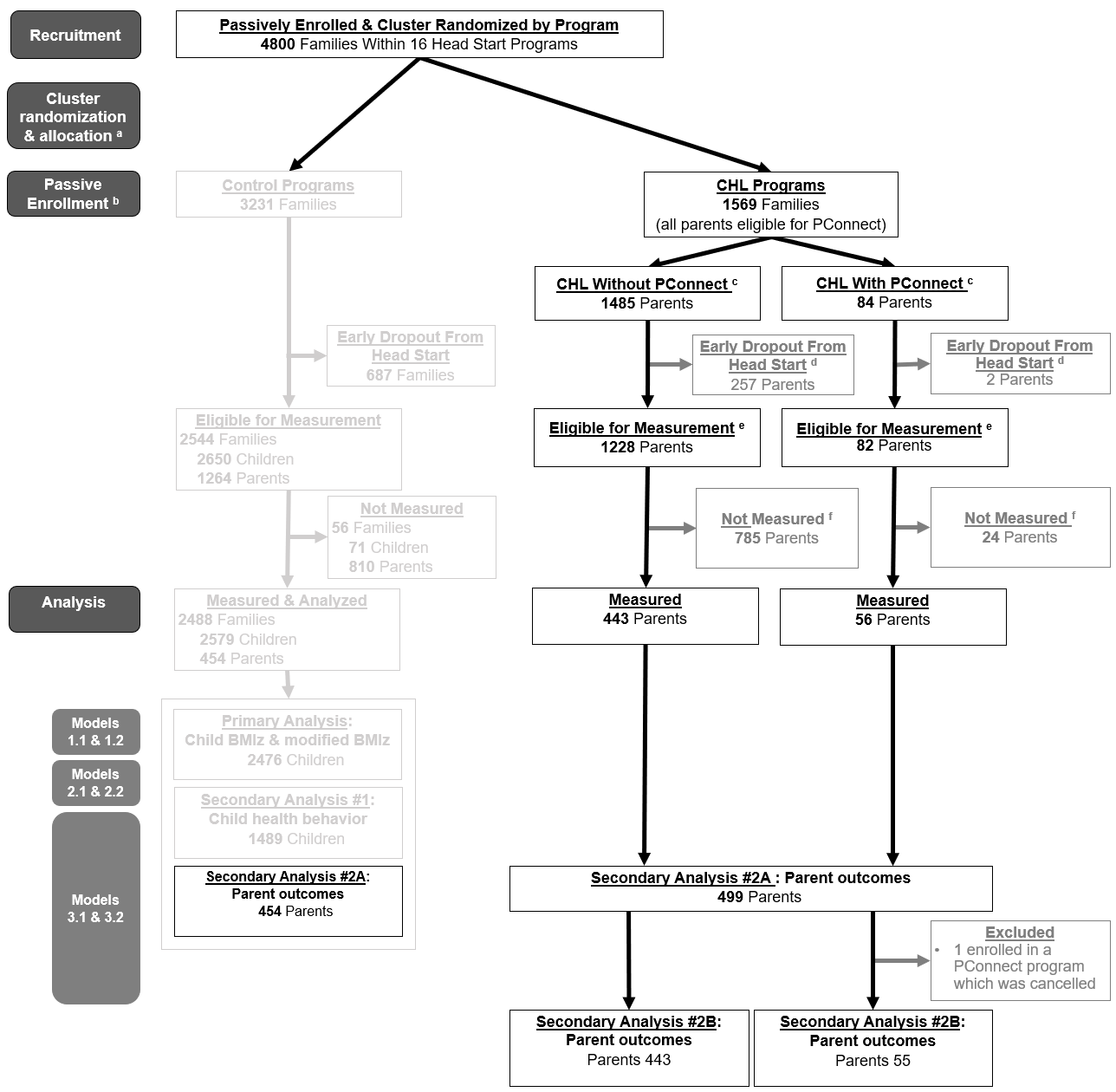
**

## **Supplemental Figure 2. Participant flow: PConnect.**

*Shown are the number of family-years (synonymous with parent-years), combined across the study period (three schoolyears, 2016-2019) and participating Head Start programs.*

***^a^*** *Allocation: Shown is the total number of family-years assigned to control and CHL programs, following cluster-randomization at the start of each schoolyear.*

***^b^*** *Enrollment: Shown is the total number of family-years enrolled at the start of the combined schoolyears.*

***^c^*** *PConnect enrollment: Total number of parents enrolled in PConnect vs. not enrolled in PConnect; enrollment defined as attending at least one of ten sessions offered. Note: 93 parents ever participated in PConnect, but eight were ineligible for analysis as they were not enrolled in Head Start at the start of the schoolyear and one was ineligible for analysis (as they were a second caregiver in the same household as another PConnect participant).*

***^d^*** *Early dropout from Head Start: Shown is the total number of parent-years who dropped out of Head Start early and did not complete the full school year.*

***^e^*** *Eligible for measurement: Shown are the total parent-years eligible for survey completion; this includes all parents who were enrolled for a full schoolyear during the study period at participating Head Start programs.*

***^f^*** *Not measured: Shown is the total number of parent-years lacking sufficient data for inclusion in the analysis. The parent outcomes analyses require at least one semester of data within a given schoolyear.*

## **Supplemental Table 2. Characteristics of Head Start parents included in the secondary analysis on parent outcomes and their children by eligibility, inclusion, and treatment arm, 2017-2019.**

|  |  | **Eligible ^a^** |  | **Included in Final Sample ^b, c^** | | | |
| --- | --- | --- | --- | --- | --- | --- | --- |
|  |  | **All**  (n=2574) |  | **All**  (n=955) | **Control**  (n=454) | **Intervention**  (n=501) | **PConnect**  (n=55) |
|  |  | % |  | % | % | % | % |
| Parent age (years) **^d^** | | 34.0 |  | 34.6 | 34.3 | 34.9 | 35.7 |
| Parent sex | |  |  |  |  |  |  |
|  | Male | 2.6 |  | 2.9 | 3.5 | 2.4 | 3.6 |
|  | Female | 96.6 |  | 96.2 | 96.0 | 96.4 | 96.4 |
|  | Missing | 0.78 |  | 0.84 | 0.44 | 1.2 | 0.0 |
| Parent race/ethnicity | |  |  |  |  |  |  |
|  | NH Asian | 10.1 |  | 13.8 | 7.1 | 20.0 | 16.4 |
|  | NH Black/AA | 34.3 |  | 32.9 | 46.9 | 20.2 | 14.6 |
|  | Hispanic/Latino | 42.3 |  | 40.8 | 36.6 | 44.7 | 60.0 |
|  | NH Other **^e^** | 2.0 |  | 2.0 | 2.0 | 2.0 | 3.6 |
|  | NH White | 8.9 |  | 9.3 | 6.6 | 11.8 | 5.5 |
|  | Missing | 2.3 |  | 1.2 | 0.88 | 1.4 | 0.0 |
| Parent spoken English proficiency | |  |  |  |  |  |  |
|  | Not at all or not well | 31.4 |  | 32.4 | 22.7 | 41.1 | 45.5 |
|  | Well or very well | 63.8 |  | 62.1 | 73.6 | 51.7 | 49.1 |
|  | Missing | 4.8 |  | 5.6 | 3.7 | 7.2 | 5.5 |
| Parent level of education attained | |  |  |  |  |  |  |
|  | < High school | 24.8 |  | 22.3 | 19.6 | 24.8 | 25.5 |
|  | High school | 37.9 |  | 37.9 | 37.7 | 38.1 | 41.8 |
|  | > High school | 34.5 |  | 36.3 | 39.2 | 33.7 | 29.1 |
|  | Missing | 2.9 |  | 3.5 | 3.5 | 3.4 | 3.6 |
| Parent employment status | |  |  |  |  |  |  |
|  | Unemployed | 24.9 |  | 25.3 | 31.3 | 20.0 | 34.6 |
|  | Employed | 23.8 |  | 24.6 | 17.8 | 30.7 | 45.5 |
|  | Other | 49.3 |  | 46.7 | 47.1 | 46.3 | 18.2 |
|  | Missing | 2.0 |  | 3.4 | 3.7 | 3.0 | 1.8 |
| Number of parents in home | |  |  |  |  |  |  |
|  | Single parent | 62.7 |  | 58.6 | 67.6 | 50.5 | 36.4 |
|  | Two parents | 37.3 |  | 41.4 | 32.4 | 49.5 | 63.6 |
| Number of children in home | |  |  |  |  |  |  |
|  | 1 | 39.4 |  | 41.3 | 40.3 | 42.1 | 25.5 |
|  | 2 | 37.3 |  | 37.9 | 36.1 | 39.5 | 47.3 |
|  | 3+ | 23.1 |  | 20.5 | 23.4 | 18.0 | 27.3 |
|  | Missing | 0.19 |  | 0.31 | 0.22 | 0.40 | 0.0 |
| Child age (months) **^d^** | |  |  |  |  |  |  |
|  | 2 years old | 11.2 |  | 10.5 | 10.8 | 10.2 | 7.3 |
|  | 3 years old | 55.1 |  | 58.4 | 61.2 | 55.9 | 63.6 |
|  | 4 years old | 33.7 |  | 31.1 | 28.0 | 33.9 | 29.1 |
| Child sex | |  |  |  |  |  |  |
|  | Male | 48.8 |  | 47.3 | 48.7 | 46.1 | 49.1 |
|  | Female | 51.2 |  | 52.7 | 51.3 | 53.9 | 50.9 |
| Child race & ethnicity | |  |  |  |  |  |  |
|  | NH Asian | 10.4 |  | 13.9 | 7.1 | 20.2 | 16.4 |
|  | NH Black/AA | 34.2 |  | 32.0 | 46.7 | 18.8 | 12.7 |
|  | Hispanic/Latino | 44.0 |  | 42.7 | 37.9 | 47.1 | 61.8 |
|  | NH Other **^e^** | 4.4 |  | 4.5 | 3.5 | 5.39 | 5.5 |
|  | NH White | 6.7 |  | 6.7 | 4.6 | 8.6 | 3.6 |

*Shown is the percent.*

***^a^*** *Parents eligible for survey completion must have a child enrolled at a participating Head Start program for a full school year in year 1 or year 2 of the study period (2017-2019).*

***^b^*** *Those included in the analysis have at least one semester of parent outcomes measured (i.e., fall and/or spring measures of a given schoolyear).*

***^c^*** *Parents who were enrolled in a cancelled PConnect program (n=2) were eligible for the first analysis, comparing parent outcomes among those exposed to intervention (n=501) vs. control (n=454); however, they were not eligible for the analysis comparing parents within the intervention arm, with PConnect (n=55) vs. without PConnect (n=443). Therefore, these two parents are included in the second column, but excluded from the fourth column. Note that the PConnect parent sample (n=55) is nested within the larger intervention sample of parents (n=501).*

***^d^*** *Age is that on September 1 of the school year enrolled.*

***^e^*** *“Other” category includes individuals who identified as biracial/multiracial, as well as American Indian, Alaska Native, Native Hawaiian or Pacific Islander.*

## **Supplemental Table 3. Sensitivity analyses for primary outcomes: Estimated mean change in BMIz and modified BMIz scores from baseline to follow-up among Head Start children (n=3750), stratified by weight status.**

|  | **Weight Status** | **Estimated change in outcome**  **(Intervention vs. control)** | |
| --- | --- | --- | --- |
|  |  | **Model 1.2 (Adjusted ^a^)** | |
| **BMIz** | Underweight | | -0.02 (-0.27, 0.24) |
|  | Healthy weight | | -0.07 (-0.14, 0.01) |
|  | Overweight | | -0.01 (-0.07, 0.06) |
|  | Obese | | **0.11 (0.05, 0.17) ***** |
| **Modified BMIz** | Underweight | | -0.01 (-0.21, 0.18) |
|  | Healthy weight | | **0.05 (0.008, 0.10) *** |
|  | Overweight | | -0.003 (-0.07, 0.07) |
|  | Obese | | **0.19 (0.08, 0.29) ***** |

*Stars indicate significance *p≤0.05, **p≤0.01, ***p≤0.001.*

*^a^ Results from a linear mixed effects regression model, adjusting for parent race and ethnicity, educational attainment, and household employment status; shown is the mean adjusted change (95% confidence interval) in outcome for children exposed to intervention relative to control.*

## **Supplemental Table 4. Frequency of parents at follow-up (n=677), by intervention status, who reported activities consistent CHL’s enhanced nutrition support and the media campaign**

|  |  |  | **Control (n=310)** |  | **CHL (n=367)** |  | **P-value***^a^* |
| --- | --- | --- | --- | --- | --- | --- | --- |
|  |  |  | **%** |  | **%** |  |  |
| **Nutrition support (intervention component 1):** Parent reports they spoke to a nutrition counselor about child’s… | |  |  |  |  |  |  |
|  | Nutrition |  | 62.9 |  | 71.1 |  | <.001*** |
|  | Physical activity |  | 54.8 |  | 66.2 |  | <.001*** |
|  | Screen time |  | 40.7 |  | 55.6 |  | <.001*** |
|  | Sleep |  | 50.7 |  | 62.1 |  | <.001*** |
|  | Sugary drink intake |  | 32.3 |  | 43.3 |  | <.001*** |
| **Media campaign (intervention component 2):** Parent reports they read brochures about child’s… | |  |  |  |  |  |  |
|  | Nutrition |  | 74.5 |  | 86.7 |  | 0.01* |
|  | Physical activity |  | 71.3 |  | 82.8 |  | 0.003** |
|  | Screen time |  | 55.8 |  | 75.2 |  | <.001*** |
|  | Sleep |  | 56.8 |  | 76.0 |  | 0.004** |
|  | Sugary drink intake |  | 57.4 |  | 71.4 |  | <.001*** |

*Shown are the frequency of parents who, in the spring semester, recall interacting with key aspects of the media campaign and/or the enhanced nutrition support. Stars indicate significance *p≤0.05, **p≤0.01, ***p≤0.001.*

*^a^Significant differences by intervention assignment (control vs. intervention) were determined from chi-squared test.*

**Supplemental Table 5. Estimated degree of heterogeneity in CHL intervention effect across study years 1 vs. 2.**

|  | **Outcome** | **Model** | **Estimate (95% CI)** | **P-Value** |
| --- | --- | --- | --- | --- |
|  |  |  |  |  |
| Corresponding with Table 5 | | | |  |
|  | BMIz | Unadjusted *^a^* | 0.04 (-0.04, 0.12) | 0.36 |
|  | BMIz | Adjusted *^b^* | 0.04 (-0.04, 0.12) | 0.36 |
|  | Modified BMIz | Unadjusted *^a^* | 0.05 (-0.03, 0.14) | 0.24 |
|  | Modified BMIz | Adjusted *^b^* | 0.05 (-0.03, 0.14) | 0.24 |
| Corresponding with Table 6 | | | |  |
|  | Fruit | Unadjusted *^a^* | -0.25 (-0.74, 0.24) | 0.32 |
|  |  | Adjusted *^b^* | -0.27 (-0.77, 0.23) | 0.28 |
|  | Juice | Unadjusted *^a^* | 0.47 (-0.31, 1.25) | 0.23 |
|  |  | Adjusted *^b^* | 0.53 (-0.26, 1.31) | 0.19 |
|  | Physical activity | Unadjusted *^a^* | -1.31 (-1.98, -0.63) | **0.0001** |
|  |  | Adjusted *^b^* | -1.42 (-2.11, -0.72) | **<.0001** |
|  | Screen | Unadjusted *^a^* | 0.31 (-0.27, 0.9) | 0.29 |
|  |  | Adjusted *^b^* | 0.21 (-0.39, 0.82) | 0.48 |
|  | Sleep duration | Unadjusted *^a^* | 0.44 (-0.12, 1.01) | 0.12 |
|  |  | Adjusted *^b^* | 0.36 (-0.21, 0.94) | 0.21 |
|  | SSB | Unadjusted *^a^* | -0.36 (-0.87, 0.15) | 0.17 |
|  |  | Adjusted *^b^* | -0.43 (-0.95, 0.1) | 0.11 |
|  | Vegetable | Unadjusted *^a^* | -0.24 (-0.82, 0.35) | 0.43 |
|  |  | Adjusted *^b^* | -0.18 (-0.79, 0.42) | 0.55 |
|  | Water | Unadjusted *^a^* | -0.66 (-1.15, -0.17) | **0.009** |
|  |  | Adjusted *^b^* | -0.73 (-1.24, -0.23) | **0.005** |
| Corresponding with Table 7 | | | |  |
|  | Empowerment | Unadjusted *^a^* | 0.02 (-0.12, 0.15) | 0.80 |
|  |  | Adjusted *^b^* | 0.03 (-0.09, 0.14) | 0.63 |
|  | Parenting | Unadjusted *^a^* | 0.02 (-0.12, 0.16) | 0.77 |
|  |  | Adjusted *^b^* | 0.04 (-0.07, 0.16) | 0.49 |
| Corresponding with Table 8 | | | |  |
|  | Empowerment | Unadjusted *^a^* | -0.25 (-0.53, 0.04) | 0.09 |
|  |  | Adjusted *^b^* | -0.27 (-0.55, 0.02) | 0.07 |
|  | Parenting | Unadjusted *^a^* | -0.14 (-0.4, 0.12) | 0.28 |
|  |  | Adjusted *^b^* | -0.15 (-0.41, 0.11) | 0.25 |
|  |  |  |  |  |

*^a^ Results from an unadjusted linear mixed effects regression model; shown is the mean unadjusted intervention by time (interaction) estimate (95% confidence interval) among those exposed to intervention relative to control, in study year 1 vs. 2.*

*^b^ Results from a linear mixed effects regression model, adjusting for parent race and ethnicity, educational attainment, and household employment status; shown is the mean adjusted intervention by time (interaction) estimate (95% confidence interval) among those exposed to intervention relative to control, in study year 1 vs. 2.*

## **Supplemental Table 6. Modeled relative mean change in parental empowerment and parenting scores among parents exposed to intervention without PConnect vs. with high or low dose PConnect**

|  |  |  | **Estimated change in outcome**  **(intervention without PConnect vs. with PConnect)** | | |
| --- | --- | --- | --- | --- | --- |
|  | **Outcome** | **PConnect Dose** | **Unadjusted** ^a^ |  | **Adjusted** ^b^ |
|  | Empowerment | Low *^c^* | -0.06 (-0.29, 0.16) |  | -0.04 (-0.27, 0.19) |
|  |  | High *^d^* | **0.29 (0.12, 0.46)***** |  | **0.28 (0.11, 0.44)***** |
|  | Parenting | Low *^c^* | -0.02 (-0.24, 0.19) |  | -0.01 (-0.21, 0.2) |
|  |  | High *^d^* | 0.1 (-0.06, 0.26) |  | 0.11 (-0.04, 0.27) |

*Stars indicate significance *p≤0.05, **p≤0.01, ***p≤0.001.*

*^a^ Results from an unadjusted linear mixed effects regression model; shown is the mean unadjusted change (95% confidence interval) in outcomes for parents exposed to intervention relative to control.*

*^b^ Results from a linear mixed effects regression model, adjusting for parent race and ethnicity, educational attainment, and household employment status; shown is the mean adjusted change (95% confidence interval) in outcomes for parents exposed to intervention relative to control.*

*^c^ Low PConnect Dose = Enrolled and participated in PConnect, but did not graduate (e.g., attended less than 70% of sessions offered)*

*^d^ High PConnect Dose = Graduated PConnect (e.g., attended at least 70% of sessions offered)*
